# Moxifloxacin induced fatal hepatotoxicity in a 72-year-old man: a case report

**DOI:** 10.4076/1757-1626-2-8063

**Published:** 2009-07-20

**Authors:** Rajanshu Verma, Radhika Dhamija, Donald H Batts, Stephen C Ross, Mark E Loehrke

**Affiliations:** Department of Internal Medicine, Michigan State University/KCMS1000 Oakland Dr, Kalamazoo, Michigan 49008USA

## Abstract

Moxifloxacin is a newer-generation synthetic fluoroquinolone that is used for treatment of acute bacterial sinusitis, acute exacerbation of chronic bronchitis, community acquired pneumonia, intra-abdominal infections and skin/skin structure infections. We describe a case of fatal hepatotoxicity caused by Moxifloxacin in a 72-year-old man. He presented with jaundice and epigastric tenderness that started one week after being treated for acute exacerbation of his chronic bronchitis with Moxifloxacin by his primary care physician. He was admitted to intensive care unit for close monitoring. His labs showed marked elevation in liver enzymes and bilirubin. His condition continued to deteriorate in intensive care unit despite supportive care. Acute hepatic failure which resulted in his death was attributed to idiosyncratic reaction to Moxifloxacin.

## Introduction

Moxifloxacin is a newer-generation (4th generation) synthetic fluoroquinolone that is used for treatment of acute bacterial sinusitis, acute exacerbation of chronic bronchitis, community acquired pneumonia, intra-abdominal infections and skin/skin structure infections [1]. It is broad spectrum and covers both gram positive and gram negative bacteria and acts by inhibiting bacterial topoisomerase II (DNA gyrase) and topoisomerase IV. Moxifloxacin is metabolized mainly in liver via glucuronide and sulfate conjugation. Cytochrome P450 system is not involved and thus there are few drug-drug interactions associated with Moxifloxacin. It has been known to cause common side effects like nausea, diarrhea, dizziness and some rare side effects like anaphylaxis, hypersensitivity reactions, nephrotoxicity, hepatotoxicity, colitis, tendon rupture, serum sickness and QTc prolongation [1]. There is very scant literature on severe hepatotoxicity caused by Moxifloxacin [2,3] and even fewer case reports of fatal hepatotoxicity caused by the same. We report a rare case of fatal hepatotoxicity caused by Moxifloxacin in a 72-year-old man.

## Case presentation

A 72-year-old Caucasian man was brought to the ER with symptoms of generalized weakness, increasing fatigue, leg swelling and jaundice for past 10 days. He was recently treated for acute exacerbation of his chronic bronchitis with steroids, bronchodilators and moxifloxacin for 5 days by his primary care physician. A week following treatment, his wife noticed yellowing of his eyes and skin. He denied any fever, sick contacts, any recent travel within or outside United States or history of insect bites. His past medical history was significant for COPD, rheumatoid arthritis and remote history of squamous cell carcinoma of the skin. Family history was significant for bone cancer, breast cancer, AAA and stroke. Patient quit smoking 40 years ago; he drinks alcohol very rarely and denied illicit drug use. His home medications included Tiotropium bromide handihaler, Salmeterol/Fluticasone inhaler, albuterol (salbutamol) inhalers, folic acid and low dose methotrexate for rheumatoid arthritis.

Patient was admitted to ICU for close monitoring due to his multiple medical comorbidities. On exam, at time of presentation his vitals were normal; he was not in acute distress. Scleral icterus was seen. His chest and cardiac exam was normal. Abdomen exam revealed mild RUQ and marked epigastric tenderness. He had bilateral pitting pedal edema. No petechiae or purpura were seen.

His labs showed a WBC of 2.6, Hb 11.2, hematocrit 32.3, platelet 13000, peripheral blood smear showed pancytopenia without atypical cells, blood cultures × 5 days were negative, LDH 169, BUN 72, Cr 2.8, Na^+^ 134, K^+^ 5.2, AST 150, ALT 193, alkaline phosphatase 337, total bilirubin 29.0, CO2 18, total protein 4.4, albumin 2.8, CRP 0.7, ESR 5, and ammonia 29. Serum lipase and amylase were normal.

Infectious diseases staff was consulted considering infection as the likely explanation. HIV, Hepatitis A, B and C panel, IgG CMV, Monospot, urine Ag for Histoplasmosis, Quantiferon TB Gold and serology for leptospira were all negative. Platelet antibody, heavy metal screen for arsenic, lead and mercury were negative. Hematology-Oncology service was consulted for pancytopenia. Serum vitamin B12, RBC folate, methylmalonic acid levels were within normal limits. Bone marrow biopsy was done and it was negative for any lymphoma/leukemia or myelophthisic process. Bacterial and fungal cultures on bone marrow were also negative.

CT scan abdomen and pelvis showed mild splenomegaly, non obstructive gallstones without cholecystitis. It was negative for masses, lymph nodes, ascites or any process suggestive of sepsis or hepatic failure.

His liver function continued to deteriorate over days despite full supportive care in ICU. His methotrexate was held in light of deranged liver enzymes. Following this extensive workup, his acute hepatic failure with blood dyscrasia was attributed to idiosyncratic reaction to Moxifloxacin. Patient died during his stay in ICU due to overwhelming multi-organ system failure.

## Discussion

More than 600 drugs have been implicated in causing liver disease. In United States, more than 20% cases of jaundice in elderly are caused by drugs. Diagnosis of drug induced liver injury (DILI) is based on finding elevated liver enzymes or development of hepatitis like symptoms, jaundice which cannot be explained due to any other cause. Most cases occur within 1 week to 3 months of exposure of drug. Liver damage may present as acute hepatocellular injury, cholestatic injury, granulomatous hepatitis, vascular insult, chronic hepatitis or neoplastic lesion [4].

Idiosyncratic drug reactions are responsible for 13% of acute liver failure in the United States. Antibiotics are the most common cause of DILI. There is 10% mortality associated with drug induced hepatocellular jaundice [5].

Liver injury is described as:

- ALT (SGPT) > 3 times upper limit of normal or
- Alkaline phosphatase > 2 times upper limit of normal or
- Total bilirubin > 2 times upper limit of normal AND increased ALT or increased alkaline phosphatase

Drug induced liver injury (DILI) categories are based on calculation of ratio of ALT to alkaline phosphatase [5]. It is subdivided as follows:

- Hepatocellular if R ≤ 5
- Cholestatic if R ≤ 2
- Mixed if 2 ≤ R ≤ 5

$$\text{where R}=\frac{\text{ALT/(upper limit normal)}}{\text{Alk Phos/(upper limit NL)}}$$

DILI is a diagnosis of exclusion. There is temporal association but no specific biochemical or histologic pattern which is pathognomonic for DILI. Drug induced liver injury shows improvement with discontinuation of the drug.

Dysfunctional Single Nucleotide Polymorphisms (SNP) have been implicated in DILI susceptibility [6-8].

Idiosyncratic reactions are rare, unpredictable and not dose dependent but can cause significant morbidity and mortality. There have been very few case reports of hepatic failure due to Moxifloxacin in literature [2,3]. As per Moxifloxacin packaging insert [1] most common side effects include nausea, vomiting, diarrhea and dizziness. Serious adverse effects being QTc prolongation (cardiotoxicity), convulsions, cartilage erosion, anaphylaxis, hypersensitivity, pseudomembranous colitis and tendon rupture. Some other adverse effects of Moxifloxacin reported in medical literature include hypersensitivity pneumonitis, immediate hypersensitivity, neutropenia, syncope, glycemic instability and arthropathy [9-15].

Our case is an important addition to the scant medical literature available in form of published case reports providing evidence on Moxifloxacin induced severe hepatotoxicity which can be fatal.

## Conclusion

Primary care and critical care clinicians should use caution in using fluoroquinolones (moxifloxacin) in patients with pre-existing liver disease or those at risk of having liver disease and being prompt in discontinuing this drug at earliest signs of liver compromise as it could result in fatal outcomes.

## Abbreviations

AAA: Abdominal Aortic Aneurysm
Ag: Antigen
ALT: Alanine Aminotransferase
AST: Aspartate Aminotransferase
BUN: Blood Urea Nitrogen
CMV: Cytomegalovirus
COPD: Chronic Obstructive Pulmonary Disease
CO2: bicarbonate
CRP: C-Reactive Protein
CT: Computer Tomography
DILI: Drug Induced Liver Injury
DNA: Deoxyribonucleic Acid
ER: Emergency Room
ESR: Erythrocyte Sedimentation Rate
HIV: Human Immunodeficiency Virus
ICU: Intensive Care Unit
Ig: Immunoglobulin
LDH: Lactate Dehydrogenase
RBC: Red Blood Cell
RUQ: Right Upper Quadrant
SGPT: Serum Glutamic Pyruvic Transaminase
SNP: Single Nucleotide Polymorphism
WBC: White Blood Cell count
